# Rosmarinic acid induces rabbit articular chondrocyte differentiation by decreases matrix metalloproteinase-13 and inflammation by upregulating cyclooxygenase-2 expression

**DOI:** 10.1186/s12929-017-0381-5

**Published:** 2017-09-18

**Authors:** Seong-Hui Eo, Song Ja Kim

**Affiliations:** Department of Biological Sciences, College of Natural Sciences, Kongju National University, Gongju, 32588 Republic of Korea

**Keywords:** Rosmarinic acid (Ros. A), Matrix metalloproteinase-13 (MMP-13), Type II collagen, COX-2, Chondrocytes, ERK pathway, p38 Kinase pathway

## Abstract

**Background:**

Matrix metalloproteinases (MMPs) are known to play an important role in the degradation of the extracellular matrix and the pathological progression of osteoarthritis (OA). The natural polyphenolic compound rosmarinic acid (Ros. A) has been shown to suppress the inhibitory activity of matrix metalloproteinases (MMPs). However, the effects of Ros. A on OA have not been investigated.

**Methods:**

In the current study, primary articular chondrocytes were cultured from rabbit articular cartilage and treated with Ros. A. Phenotypic characterization was performed by western blotting to assess specific markers, prostaglandin E_2_ (PGE_2_) assays, and alcian blue staining to measure sulfated-proteoglycan production.

**Results:**

We report that in rabbit articular chondrocytes, Ros. A increased type II collagen, sulfated-proteoglycan, cyclooxygenase-2 (COX-2), and PGE_2_ production in a dose- and time-dependent manner. Furthermore, Ros. A suppressed the expression of MMP-13. In addition, treatment with Ros A activated extracellular signal-regulated kinase (ERK)-1/2 and p38 kinase signaling pathways. Inhibition of MMP-13 enhanced Ros. A-induced type II collagen expression and sulfated-proteoglycan synthesis but COX-2 and PGE_2_ production were unchanged. Ros. A-mediated up-regulation of ERK phosphorylation was abolished by the MEK inhibitor, PD98059, which prevented induction of the associated inflammatory response. Inhibition of p38 kinase with SB203580 enhanced the increase in type II collagen expression via Ros. A-mediated down-regulation of MMP-13.

**Conclusions:**

Results suggest that ERK-1/2 regulates Ros. A-induced inflammation and that p38 regulates differentiation by inhibiting MMP-13 in rabbit articular chondrocytes.

## Background

Osteoarthritis (OA), the most common chronic and degenerative articular disease, is most prevalent in the elderly [1, 2]. The main feature of this disease is cartilage degradation, formation of osteophytes, and synovial inflammation, among other alterations. The cause of OA is still unclear, and there is no disease-modifying treatment available except for joint replacement surgery [3].

Articular chondrocytes are the only resident cell type in articular cartilage, which is composed of dense collagenous extracellular matrix (predominantly type II) and proteoglycans (importantly aggrecan) [4]. In articular cartilage, the loss of type II collagen and proteoglycans is due to the activation of extracellular matrix (ECM)-degrading enzymes such as matrix metalloproteinases (MMPs) and ADAMTS [5]. In particular, MMPs play a central role in articular cartilage destruction in OA and rheumatoid arthritis patients. Among them, MMP-13 was reported to play important roles in the degradation of proteoglycans and the activation of pro-collagenase in the cartilage of individuals with OA [5, 6].

In addition to MMPs, cyclooxygenase-2 (COX-2) and inducible nitric oxide synthases also play important roles in the pathogenesis of OA [7]. COX enzymes metabolize arachidonic acid promoting the formation of prostaglandin H2, which leads to increased production of prostaglandin E2 (PGE_2_). There are two isoforms of the COX enzyme: COX-1 is found in most tissues and is constitutively expressed in normal cells, whereas COX-2 is not expressed in healthy tissue but is stimulated by inflammatory cytokines (interleukin-1 beta (IL-1β) and tumor necrosis factor (TNF-α)), growth factors, and tumor promoters [8–11]. Moreover, COX-2 and PGE_2_ are important mediators of inflammation and are implicated in bone resorption and joint pain [10].

Rosmarinic acid (Ros. A; α-*o*-caffeoyl-3,4-dihydroxyphenyl lactic acid) a naturally phenolic compound that is mainly found in species of the Boraginaceae, Labiate, and Anthocerotaceae families of herbs [12, 13], and was first isolated from rosemary (*Rosmarinus officinalis*). Many studies have reported that Ros. A has various biological and pharmacological activities, such as anti-tumor, anti-oxidant, anti-inflammatory, anti-fibrosis, anti-mutagenic, and hepatoprotective effects [13–15]. Recently, studies have examined the inhibitory effect of Ros. A on MMP-2 and MMP-13 activity [16, 17]. Hence, Ros. A is used to treat bronchial asthma, peptic ulcers, cataracts, arthritis, cancer, and rheumatoid arthritis [14]. However, the molecular mechanism underlying Ros. A-induced inflammation and differentiation, mediated by MMP-13 expression, has not been elucidated.

The mitogen-activated protein kinase (MAPK) is a signaling pathway that is can be activated in articular cartilage; it is well documented that this pathway regulates the production of MMPs, which are devoted to the degeneration of chondrocytes [18]. MAPK family members include extracellular signal-regulated kinase (ERK)-1/2, p38, and c-Jun N-terminal kinase (JNK). ERK-1/2 and p38 play major roles in mediating chondrocyte proliferation and differentiation and have been found to be associated with inflammation [18, 19].

In this study, we investigated if Ros. A can regulate differentiation and inflammation in chondrocytes. Our findings indicate that Ros. A promotes the inflammatory response of rabbit articular chondrocytes by activating ERK and suppressing MMP-regulated differentiation via the p38 kinase pathway.

## Methods

### Reagents and antibodies

Rosmarinic acid (Ros. A), with purity greater than 98%, was purchased from Cayman chemical Co. (Ann Arbor, MI, USA). Primary antibodies specific for MMP-13, type II collagen, and actin were obtained from Santa Cruz Biotechnology Inc. (Santa Cruz, CA, USA) and phosphorylated ERK and phosphorylated p38 MAP kinase antibodies were purchased from Cell Signaling Technology Inc. (Danvers, MA, USA). A COX-2 antibody was obtained from Cayman chemical Co. Anti-rabbit IgG antibody and anti-goat lgG were obtained from Sigma-Aldrich (St. Louis, MO, USA) and anti-mouse IgG was purchased from Enzo Life Sciences International, Inc. (New York, NY, USA). Anti-mouse IgG-FITC, Anti-mouse IgG-TRITC and alcian blue solution were purchased from Sigma-Aldrich.

### Primary culture of chondrocytes from rabbit articular cartilage

Rabbit articular chondrocytes were isolated from two-week-old New Zealand White rabbits (Koatech, Pyeongtaek, Republic of Korea) as described previously [20]. Cartilage slices were digested with 0.2% collagenase type II for 8 h in a 37 °C CO_2_ incubator. Isolated chondrocytes (2 × 10^5^ cells/dish) were seeded in Dulbecco’s modified Eagle’s medium (DMEM; Invitrogen, Carlsbad, CA, USA) containing 10% (*v*/v) fetal bovine serum (Tissue Culture Biologicals, Los Alamitos, CA, USA), penicillin (50 unit/mL, Sigma-Aldrich), and streptomycin (50 μg/mL, Sigma-Aldrich), and maintained as monolayers in a 5% CO_2_ incubator at 37 °C. The medium was replaced with fresh medium 2 d after seeding. This study protocol was approved by the Ethics Committee of the Kongju National University.

### Treatment of cells with Ros. A

Ros. A was first dissolved in dimethyl sulfoxide (DMSO, Sigma-Aldrich, St. Louis, USA) and then dissolved with specific culture medium to the desired final concentration; for this, the overall DMSO concentration was less than 0.1% (*v*/v). After 3 d, the cell cultures were treated with various concentrations (0, 25, 50, 75, and 100 μg/mL) of Ros. A for 3 or 24 h. Alternatively, cells were treated with 75 μg/mL Ros. A for various time periods. PD98059 (PD; Calbiochem, San Diego, CA, USA) and SB203580 (SB; Biomol, Plymouth Meeting, PA, USA), used to inhibit MMP and MMP-13, were added 2 h before treatment with Ros A. These compounds were used to inhibit ERK-1/2 and p38, respectively. The differentiation status and inflammation responses of articular chondrocytes was determined by examining the expression of type II collagen and COX-2 by western blot analysis.

### Western blot analysis

After the indicated treatment, cell samples were washed once with cold phosphate-buffered saline (PBS) and lysed using a buffer containing 50 mM tris-HCl (pH 7.4), 150 mM NaCl, 1% nonidet P-40, and 0.1% SDS supplemented with protease inhibitors and phosphatase inhibitors on ice for 30 min. Proteins were extracted and cell debris was removed by centrifugation at 13,000 rpm for 10 min at 4 °C. Protein concentrations were determined using the bicinchoninic acid (BCA) assay. Equal amounts of the extracted proteins (30 μg) were separated by 8% sodium dodecyl sulfate polyacrylamide gel electrophoresis and transferred to a nitrocellulose membrane. The membrane was blocked with 5% non-fat dry milk in tris-buffered saline (TBS) for 1 h at room temperature and washed three times with TBS containing 0.05% tween-20 (TBS-T buffer). The membrane was incubated with primary antibodies overnight at 4 °C. Membranes were then washed three times with TBS-T buffer and then incubated with peroxidase-conjugated secondary antibody for 2 h at room temperature. The enhanced chemiluminescence (ECL) reagent was used to identify reactive bands. Finally, the bands were quantified using the LAS4000 (Fuji Film, Tokyo, Japan). The bands were quantified by densitometric analysis using the ImageJ software package.

### Alcian blue staining

The cells were fixed with 3.5% paraformaldehyde in PBS at room temperature for 20 min and stained with 0.1% alcian blue in 0.1 M HCl overnight. The chondrocytes were washed three times with PBS buffer and then incubated in 6 M guanidine HCl for 6 h. Production of sulfated proteoglycan was measured at 595 nm using a microplate reader.

### PGE_2_ assay

Cells (2 × 10^4^ cells/well) were seeded in 96-well plates. After 24 h of treatment, conditioned medium was harvested and PGE_2_ concentrations were determined using an ELISA assay kit according to instructions supplied by the manufacturer (Assay Designs, Ann Arbor, MI, USA). Samples were assayed in triplicate for each of three independent experiments. PGE_2_ levels were calculated by comparing values to a standard curve.

### Immunofluorescence staining

The expression of type II collagen and COX-2 at the protein level in rabbit chondrocytes was analyzed by Immunofluorescence microscopy. Cells were fixed with 3.5% paraformaldehyde in PBS for 20 min at room temperature and permeabilized with 0.1% triton X-100 in PBS for 15 min. The cells were then blocked with 5% skim milk to prevent non-specific reactions. Then, fixed cells were incubated with antibodies against type II collagen (1:100) and COX-2 (1:100) for 2 h at room temperature. Cells were washed three times with PBS and incubated with secondary antibodies (1:50) at room temperature for 1 h. Then cells were counterstained with 4′6′-diamidi-no-2-phenylindole dihydrochloride (DAPI; Invitrogen, Burlington, ON, Canada). Fluorescence images were recorded using a BX51 fluorescence microscope (Olympus, Tokyo, Japan).

### Statistical analysis

All experimental data were replicated at least three times. Data are presented as the mean ± standard deviation (SD). Results were analyzed using a one-way analysis of variance (ANOVA), and all pairwise comparisons between groups were conducted using the Turkey post hoc test; *p* values ≤0.05 were considered statistically significant.

## Results

### Effect of Ros. A on rabbit chondrocyte differentiation

**Fig. 1:**
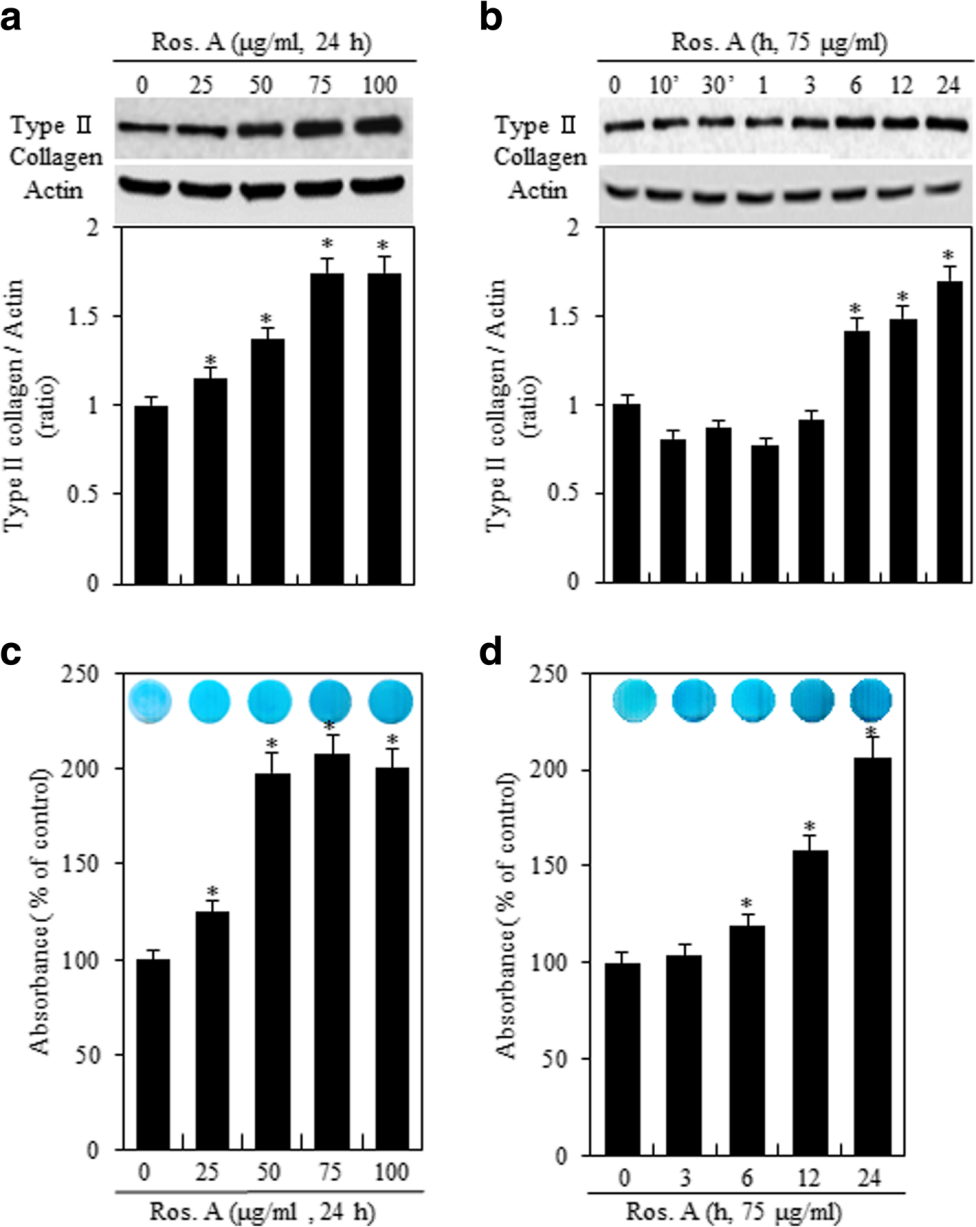
Ros. A induces differentiation in rabbit articular chondrocytes. Rabbit primary cultured articular chondrocytes were treated with or without the indicated concentrations of rosmarinic acid (Ros. A) for 24 h (**a** and **c**) or with 75 μg/mL Ros. A for the specified time periods (**b** and **d**). **a** and **b** The expression of type II collagen and actin was analyzed by western blotting (*upper panel*). Actin was used as a loading control. The relative amounts of type II collagen were quantified by densitometry measurements (Image J) (*lower panel*). **c** and **d** The synthesis of sulfated-proteoglycan was detected via alcian blue staining. The results represent three independent experiments. Values shown are the means ± SD; *p < 0.05 vs. control cells

We performed western blot analysis and alcian blue staining to identify the effects of Ros. A on the differentiation of rabbit articular chondrocytes; we examined type II collagen (a marker of chondrocyte differentiation) expression and sulfated-proteoglycan (cartilage-specific marker molecule) production after exposure to Ros. A. As shown in Fig. 1, western blot analysis showed that Ros. A increased the expression of type II collagen in a dose- and time-dependent manner (Fig. 1a and b, *upper panel*). Densitometric evaluation of western blots was performed in triplicate (Fig. 1a and b, *lower panel*). The synthesis of sulfate proteoglycans was also examined. Consistent with the expression patterns of type II collagen, alcian blue staining revealed that Ros. A induced the production of sulfated proteoglycan in a dose- and time- dependent manner (Fig. 1c and d). These results indicate that Ros. A promotes the differentiation of chondrocytes.

### Effect of Ros. A on inflammation in rabbit chondrocytes

**Fig. 2:**
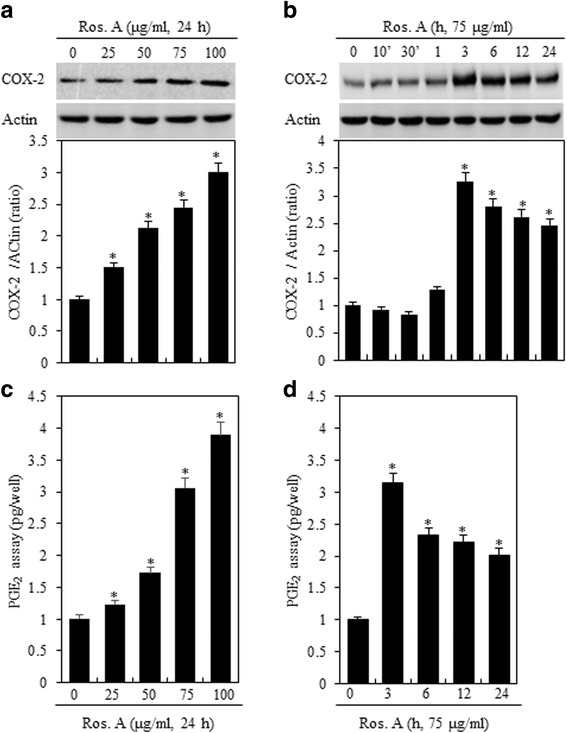
Ros. A regulates the inflammatory response in rabbit articular chondrocytes. Rabbit primary cultured articular chondrocytes were treated with or without the indicated concentrations of rosmarinic acid (Ros. A) for 24 h or 75 μg/mL Ros. A for the specified time periods. **a** and **b** Expression levels of COX-2 were determined by western blotting (*upper panel*). Actin was used as a loading control. The relative amounts of COX-2 were quantified by densitometry measurements (Image J) (*lower panel*). **c** and **d** Prostaglandin E_2_ (PGE_2_) production was measured using a PGE_2_ assay kit. The results represent three independent experiments. Values shown are the means ± SD: *p < 0.05 vs. control cells

To determine whether Ros. A affects inflammation in chondrocytes, these cells were treated with various concentrations of Ros. A for 3 h or with 75 μg/ml of Ros. A for various time periods (Fig. 2). A concentration-dependent increase in COX-2 expression was observed (Fig. 2b, *upper panel*). Stimulation of cells resulted in a marked increase in COX-2 expression, which reached a maximum at 3 h, and decreased thereafter (Fig. 2a, *upper panel*). Densitometric evaluation of representative western blots was performed in triplicate (Fig. 2a and b, *lower panel*).

To assess the effect of Ros. A on COX-2 activity, we quantified the production of PGE_2_ in untreated rabbit chondrocytes or those treated with Ros. A (Fig. 2c and d). A significant increase in PGE_2_ synthesis was verified in treated rabbit chondrocytes. Increases in PGE_2_ production and COX-2 expression induced by Ros. A were similar (Fig. 2c and d). These data suggest that Ros. A induces inflammation by increasing COX-2 expression and PGE_2_ production in rabbit articular chondrocytes.

### Effect of Ros. A on MMP-13 expression in rabbit chondrocytes

**Fig. 3:**
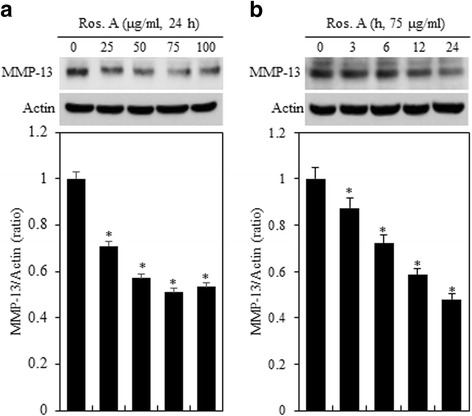
Ros. A reduces MMP-13 expression in rabbit articular chondrocytes. Rabbit primary cultured articular chondrocytes were treated with the indicated concentrations of rosmarinic acid (Ros. A) for 24 h or 75 μg/mL Ros. A for the indicated time periods. **a** and **b** Expression of MMP-13 and actin was detected by western blot analysis. Actin was used as the loading control (*upper panel*). The relative amounts of MMP-13 were quantified by densitometry measurements (Image J) (*lower panel*). The results represent three independent experiments. Values shown are the means ± SD; *p < 0.05 vs. control cells

Because previous studies have showed that Ros. A modulates the expression of MMP-13 [16], we performed western blotting to evaluate this (Fig. 3). Treatment with Ros.A resulted in a significant decrease in MMP-13 in a dose- and time- dependent manner (Fig. 3a and b, *upper panel*). Densitometric evaluation of representative western blots was performed in triplicate (Fig. 3a and b, *lower panel*).

**Fig. 4:**
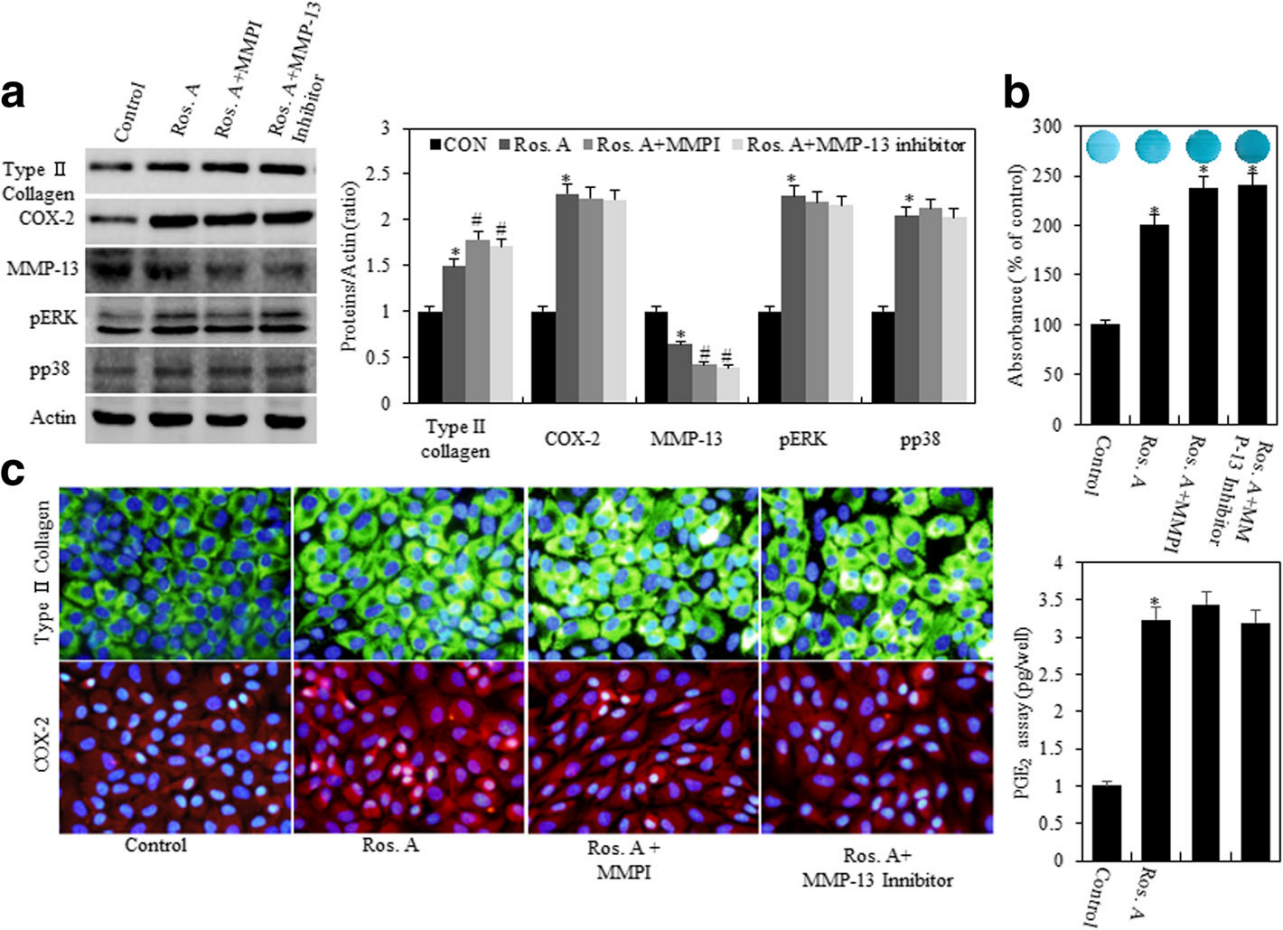
Ros. A induces differentiation by modulating MMP-13. Primary chondrocytes were left untreated or were treated with 75 μg/mL rosmarinic acid (Ros. A) in the absence or presence of 10 μM MMPI or 113 nM MMP-13 inhibitors for 24 h. **a** Type II collagen, COX-2, MMP-13, pERK, pP38, and actin were detected by western blotting. Actin was used as a loading control (*left pane*l). The relative amounts of proteins (Type II collagen, COX-2, MMP-13. pERK, pp38) were quantified by densitometry measurements (Image J) (*right panel*). **b** Sulfated proteoglycan synthesis was detected by alcian Blue staining (*upper panel*). Prostaglandin E_2_ (PGE_2_) production was measured using a PGE_2_ assay kit (*lower panel*). **c** Type II collagen and COX-2 expression was detected by immunocytochemistry. The results represent three independent experiments. Values shown are the means ± SD; *p < 0.05 compared to the control; # < 0.05 compared to the Ros. A

Many studies have shown that MMPs are involved in both physiological collagen turnover in articular cartilage and matrix degradation in OA cartilage. In addition, previous studies have suggested that inflammatory cytokines stimulate the expression of MMPs [21]. Hence, we evaluated if the aforementioned effects of Ros. A on type II collagen and COX-2 expression were because of MMP-13 activity. Chondrocytes were treated with 75 μg/mL Ros. A in the absence or presence of 10 μM MMP inhibitor (MMPI) or 113 nM MMP-13 inhibitor for 24 h (Fig. 4). Treatment with MMPI and MMP-13 inhibitors enhanced Ros. A-induced differentiation and suppressed MMP-13 expression. However, this did not affect Ros A-induced COX-2, ERK-1/2, and p38 expression (Fig. 4a, *left pane*l). Densitometric evaluation of representative western blots was performed in triplicate (Fig. 4a, *right panel*). This was confirmed by alcian blue staining, PGE_2_ assays, and immunofluorescence staining (Fig. 4b and c). These results indicate that Ros. A suppresses MMP-13-regulated type II collagen expression and sulfate proteoglycan production in rabbit articular chondrocytes. Taken together, our results suggest that RosA reduces-MMP13 regulated differentiation in rabbit articular chondrocytes.

### Effect of ERK-1/2 and p38 on Ros. A-induced differentiation and inflammation in rabbit chondrocytes

**Fig. 5:**
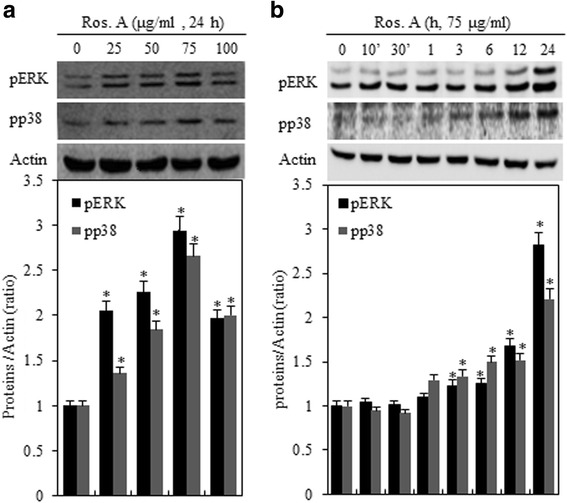
Ros. A activates the ERK-1/−2 and p38 pathway in rabbit articular chondrocytes. Cells were treated with 75 μg/mL rosmarinic acid (Ros. A) for the indicated times or with various concentration of Ros. A for 24 h. **a** and **b** ERK-1/−2, p38, and actin levels were evaluated by western blotting. Actin was used as a loading control (*upper panel*). The relative amounts of pERK and pp38 were quantified by densitometry measurements (Image J) (*lower panel*). The results represent three independent experiments. Values shown are the means ± SD; *p < 0.05 vs. control cells

**Fig. 6:**
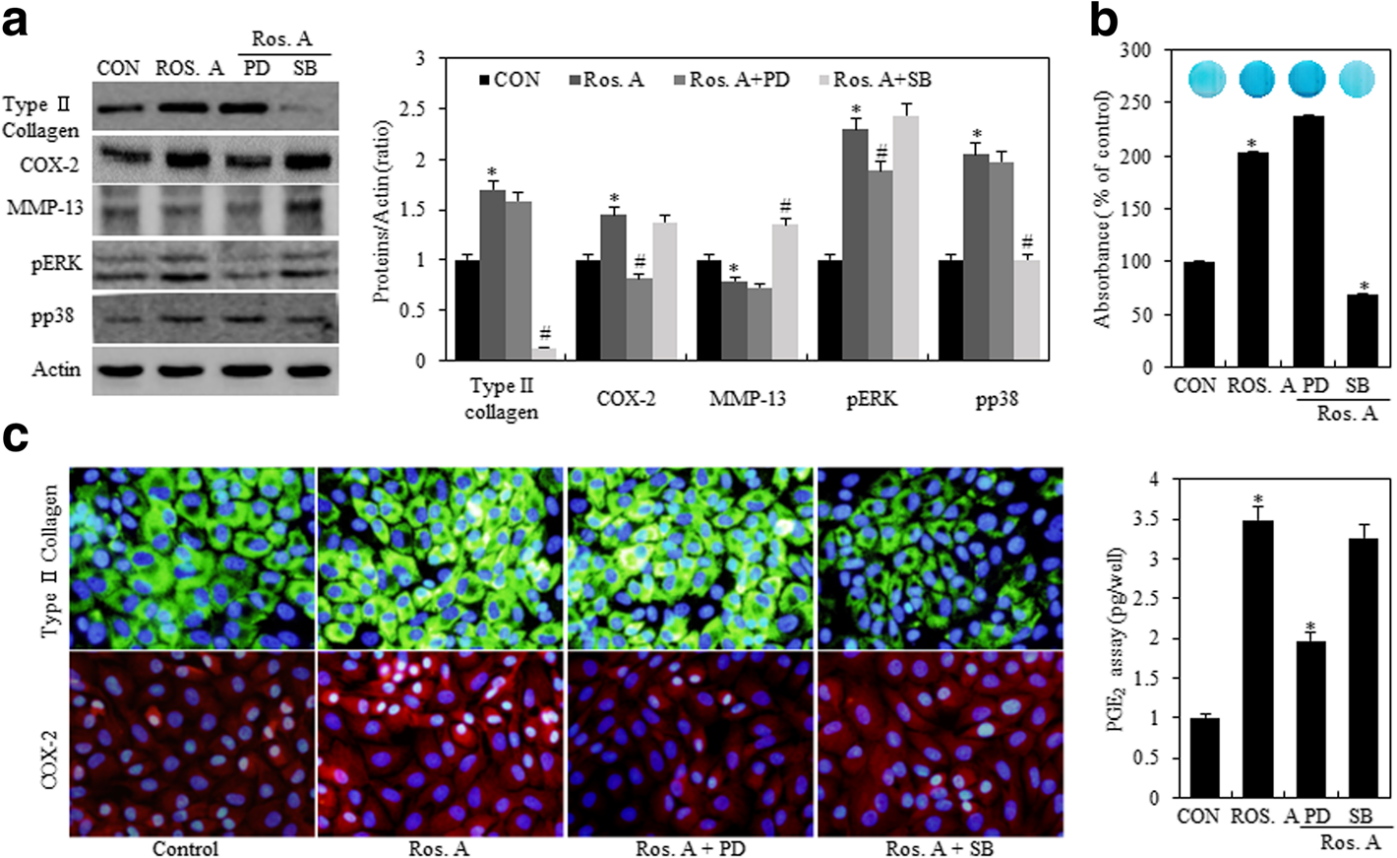
Ros. A induces differentiation and inflammatory response by p38 and ERK-1/−2 pathways. Chondrocytes were exposed to 75 μg/mL rosmarinic acid (Ros. A) in the absence or presence of 20 μM PD98059 (PD) or 20 μM SB203580 (SB) for 24 h. **a** Type II collagen, COX-2, MMP-13, pERK, pP38, and actin were detected by western blotting. Actin was used as a loading control (*left pane*l). The relative amounts of proteins (Type II collagen, COX-2, MMP-13. pERK, pp38) were quantified by densitometry measurements (Image J) (*right panel*). **b** Production of sulfate proteoglycan was determined by alcian blue staining (*upper panel*). Synthesis of prostaglandin E_2_ (PGE_2_) was analyzed by PGE_2_ assays (*lower panel*). **c** Expression of type II collagen and COX-2 was detected by immunocytochemistry. The results represent three independent experiments. Values shown are the means ± SD; *p < 0.05 compared to the control; # < 0.05 compared to the Ros. A

We next examined whether Ros. A-mediated MAPK activation is associated with the differentiation and inflammation of chondrocytes (Fig. 5). Several studies have indicated that the MAPK pathway is involved in regulating these processes [18, 19]. Western blot analysis indicated a concentration-independent increase in ERK-1/2 and p38 phosphorylation levels, reaching a maximum at 75 μg/mL (Fig. 5a, *upper panel*). Ros. A treatment resulted in the phosphorylation of p38 and ERK-1/2 in time-dependent manner (Fig. 5b, *upper panel*). Densitometric evaluation of representative western blots was performed in triplicate (Fig. 5a and b, *lower panel*). To examine whether ERK-1/−2 and p38 play crucial roles in the regulation of type II collagen and COX-2, we treated Ros. A chondrocytes with the specific inhibitors, PD or SB (Fig. 6). Treatment with the ERK-1/2 inhibitor PD restored COX-2 expression and PGE_2_ production in Ros.A-treated chondrocytes (Fig. 6a and b (*lower panel*)). Inhibition of p38 with SB promoted Ros. A-induced differentiation via MMP-13 (Fig. 6a and b (*upper panel*)). Moreover, consistent with western blotting data, immunofluorescence analysis showed that treatment with SB enhances the Ros. A-mediated increase in type II collagen, and that PD abolishes the increase in COX-2 expression (Fig. 6c).

## Discussion

OA is the most prevalent disease of joints in elderly patients, affecting many millions of individuals worldwide and resulting in knee pain and locomotor system disability [5, 22]. OA is characterized by intra-articular inflammation, cartilage degeneration, and subchondral bone remodeling. In normal physiological conditions, homeostasis is maintained in the ECM of articular chondrocytes through the regulation of synthesis and degradation [23]. However, this cartilage homeostasis is disrupted in OA and RA, causing continual loss of cartilaginous tissues [23, 24]. Much is known about how cartilage is formed during skeletal development and a number of factors are known to contribute to the development of OA. However, the exact causes of this disease are still unclear and very little is known about how to successful apply this knowledge to control the OA disease process [25, 26]. Recently, plant-derived compounds that protect or stimulate healing of the cartilage have been investigated as ideal drugs for OA, due to their few side effects and anti-inflammatory activities [27].

Type II collagen is the main protein component of and is specific for cartilage, forming up to 50% of its ECM. Particularly, this marker can be used as a factor to predict the response to therapy in OA. Collagen and proteoglycans are degradation and lost due to excessive secretion of MMPs and other proteolytic enzymes [22, 28, 29]. The MMP family contains approximately 28 members, which can be divided into subgroups such as collagenases, gelatinases, and stromelysins. Among them, MMP-1 and MMP-13 are considered to be the major collagenases in chondrocytes during conditions of low inflammation [23, 30]. Notably, MMP-13 has 5- to 10-fold greater activity than collagen type II and gelatinase activity that is greater than 44-fold higher than that of MMP-1 [31]. Therefore, MMP-13 has been considered a key regulator of cartilage degradation and has become a valid target for OA therapy. In this study, we found that Ros. A can suppress MMP-13 protein expression (Fig. 3) and the up-regulation of type II collagen and sulfated proteoglycan (Fig. 1). In addition, MMPI and MMP-13 inhibitors selectively reduced MMP-13 levels and stimulated Ros A-induced differentiation (Fig. 4).

Previous studies have provided clear evidence that OA is associated with increased production of IL-1β, which plays a key role in chondrocyte damage through the up-regulation of pro-inflammatory factors including MMPs and COX-2 [32]. Excess induction of COX-2 expression leads to elevated production of PGE_2_. Although OA tissues can be damaged by very low levels of pro-inflammatory cytokines, through increased production of MMP-13, which induces proteoglycan degradation, this was shown to further enhance the loss of type II collagen in OA joints [33]. In this study, our data demonstrated Ros. A-induced expression of COX-2 in rabbit articular chondrocytes (Fig. 2); however, degradation of type II collagen and MMP-13 expression were not elevated by COX-2. In addition, inhibition of MMP-13 by MMPI or an MMP-13 inhibitor had no effect of COX-2 expression (Fig. 4).

Ros. A was isolated for the first time from *Rosmarinus officinalis L.* by Scarpati and Oriente in 1958 [34]. This compound was structurally characterized as an ester of caffeic acid and 3,4-dihydroxyphenyllactic acid. It is known to exhibit various pharmacological activities, notably anti-oxidant, anti-microbial, and anti-inflammatory activities, and thus has been used to treat peptic ulcers, arthritis, cataracts, cancer, and bronchial asthma, among other illnesses [35]. Hur et al. reported that Ros. A induces the preferential apoptotic activity of activated and effector T-cells via the mitochondrial pathway [36]. Furthermore, Han et al. investigated the effect of RA on MKN45 human gastric cancer cells and found that it exerted an anti-cancer effect via the inhibition of pro-inflammatory cytokines and the inactivation of inflammatory pathways [15, 37]. Moon et al. reported that Ros. A treatment sensitizes human leukemia U937 cells to TNF-α-induced apoptosis through the suppression of nuclear factor-κB and reactive oxygen species [38]. In previous investigations, pretreatment with Ros. A was shown to reduce COX-2 mRNA expression in a TPA-challenged skin mouse model [39]. In addition, in a murine collagen induced arthritis model, Ros. A was shown to remarkably reduce the frequency of COX-2-expressing cells, when compared to that in untreated mice [40]. However, strikingly, Ros. A did not reduce COX-2 expression, but rather upregulated type II collagen and sulfated proteoglycan in chondrocytes.

**Fig. 7:**
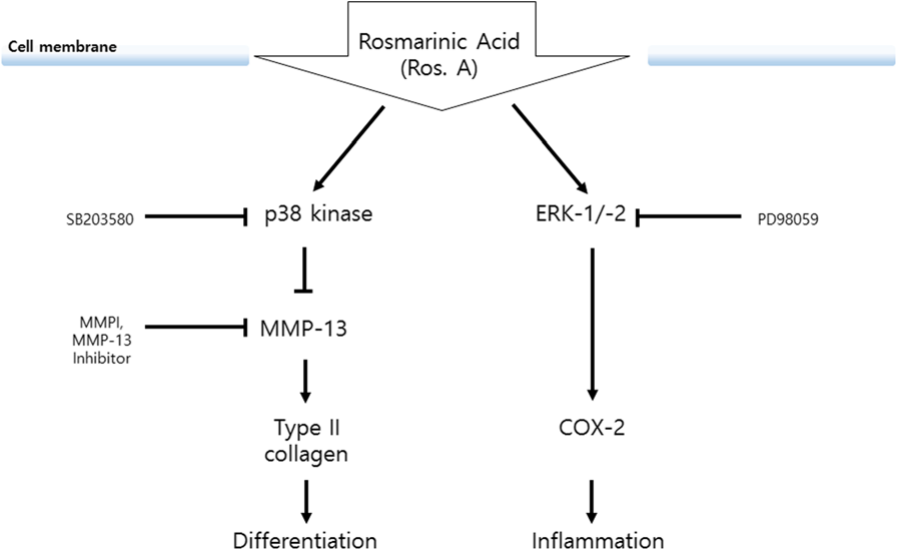
A graphical depiction of the effects of rosmarinic acid (Ros. A) on the regulation of inflammation and differentiation in rabbit articular chondrocytes

The MAPK signal transduction pathway promotes cell proliferation, differentiation, and apoptosis, which could account for the effects observed in some degenerative diseases such as OA [41, 42]. It also serves as the predominant system that regulates the production of MMPs, which promote the degeneration of chondrocytes. p38 and ERK play major roles in mediating chondrocyte proliferation, dedifferentiation, inflammation, and related gene expression [30]. To investigate the involvement of the MAPK cascade in the Ros. A-induced differentiation and inflammation of chondrocytes, the phosphorylation patterns of the ERK1−/2 and p38 were assessed by western blotting after Ros A treatment. Chondrocytes treated with Ros A displayed enhanced ERK-1/2 and p38 kinase activity (Fig. 5). Additionally, whereas inhibition of ERK, through treatment with PD, abolished Ros. A-induced COX-2 expression, suppression of p38 through treatment with SB accelerated MMP-13-induced type II collagen expression (Fig. 6). Thus, in rabbit articular chondrocytes, Ros. A enhances inflammation through ERK-1/2 signaling and MMP-regulated differentiation via MMP-13 inhibition and downstream p38 kinase signaling. A graphical pathway summarizing the underlying mechanisms is shown in Fig. 7.

## Conclusions

Our results, using Ros. A, demonstrate that that p38 regulates differentiation by inhibiting MMP-13 and that ERK-1/2 regulates Ros. A-induced inflammation in rabbit articular chondrocytes. This information is useful to understanding the molecular mechanism of OA and Ros. A may be a potential candidate for further investigation for future use in the treatment or cartilage-related disorders including OA.

## Abbreviations

COX-2: Cyclooxygenase-2
EMC: Extracellular matrix
ERK: Extracellular signal-regulated kinase
MAPK: Mitogen-activated protein kinase
MMPI: Matrix metalloproteinases inhibitor
MMPs: Matrix metalloproteinases
OA: Osteoarthritis
PD: PD98059
PGE_2_: Prostaglandin E_2_
Ros. A: Rosmarinic acid
SB: SB203580

## References

[1] Lou Y, Wang C, Zheng W, Tang Q, Chen Y, Zhang X, Guo X, Wang J (2017). Salvianolic acid B inhibits IL-1β-induced inflammatory cytokine production in human osteoarthritis chondrocytes and has a protective effect in a mouse osteoarthritis model. Int Immunopharmacol.

[2] Wang CC, Guo L, Tian FD, An N, Luo L, Hao RH, Wang B, Zhou ZH (2017). Naringenin regulates production of matrix metalloproteinases in the knee-joint and primary cultured articular chondrocytes and alleviates pain in rat osteoarthritis model. Braz J Med Biol Res.

[3] McCulloch K, Litherland GJ, Rai TS (2017). Cellular senescence in osteoarthritis pathology. Aging Cell.

[4] Findlay DM, Atkins GJ (2014). Osteoblast-Chondrocyte interactions in osteoarthritis. Curr Osteoporos Rep.

[5] Kang DG, Lee HJ, Kim KT, Hwang SC, Lee CJ, Park JS (2017). Effect of oleanolic acid on the activity, secretion and gene expression of matrix metalloproteinase-3 in articular chondrocytes in vitro and the production of matrix metalloproteinase-3 in vivo. Korean J Physiol Pharmacol..

[6] Nam DC, Kim BK, Lee HJ, Shin HD, Lee CJ, Hwang SC (2016). Effects of prunetin on the proteolytic activity, secretion and gene expression of MMP-3 in vitro and production of MMP-3 in vivo. Korean J Physiol Pharmacol.

[7] Jeong JW, Lee HH, Lee KW, Kim KY, Kim SG, Hong SH, Kim GY, Park C, Kim HK, Choi YW, Choi YH (2016). Mori folium inhibits interleukin-1β-induced expression of matrix metalloproteinases and inflammatory mediators by suppressing the activation of NF-κB and p38 MAPK in SW1353 human chondrocytes. Int J Mol Med.

[8] Cho HS, Walker A, Williams A, Hasty KA (2015). Study of osteoarthritis treatment with anti-inflammatory drugs: Cyclooxygenase-2 inhibitor and steroids. Biomed Res Int.

[9] Eo SH, Kim DW, Choi SY, Kim HA, Kim SJ (2015). PEP-1-SIRT2 causes dedifferentiation and COX-2 expression via the MAPK pathways in rabbit articular chondrocytes. Exp Cell Res.

[10] Fu Y, Lei J, Zhuang Y, Zhang K, Lu D (2016). Overexpression of HMGB1 A-box reduced IL-1β-induced MMP expression and the production of inflammatory mediators in human chondrocytes. Exp Cell Res.

[11] Ding QH, Cheng Y, Chen WP, Zhong HM, Wang XH (2013). Celastrol, an inhibitor of heat shock protein 90β potently suppresses the expression of matrix metalloproteinases, inducible nitric oxide synthase and cyclooxygenase-2 in primary human osteoarthritic chondrocytes. Eur J Pharmacol.

[12] Ellis BE, Towers GH (1970). Biogenesis of rosmarinic acid in Mentha. Biochem J.

[13] Runtuwene J, Cheng KC, Asakawa A, Amitani H, Amitani M, Morinaga A, Takimoto Y, Kairupan BH, Inui A (2016). Rosmarinic acid ameliorates hyperglycemia and insulin sensitivity in diabetic rats, potentially by modulating the expression of PEPCK and GLUT4. Drug Des Devel Ther..

[14] Hajhosseini L, Khaki A, Merat E, Ainehchi N (2013). Effect of rosmarinic acid on sertoli cells apoptosis and serum antioxidant levels in rats after exposure to electromagnetic fields. Afr J Tradit Complement Altern Med.

[15] Cao W, Hu C, Wu L, Xu L, Jiang W (2016). Rosmarinic acid inhibits inflammation and angiogenesis of hepatocellular carcinoma by suppression of NF-κB signaling in H22 tumor-bearing mice. J Pharmacol Sci.

[16] Ao C, Li A, Elzaawely AA, Tawata S (2008). MMP-13 inhibitory activity of thirteen selected plant species from Okinawa. Int J of Pharm.

[17] Murata T, Sasaki K, Sato K, Yoshizaki F, Yamada H, Mutoh H, Umehara K, Miyase T, Warashina T, Aoshima H, Tabata H, Matsubara K (2009). Matrix metalloproteinase-2 inhibitors from Clinopodium Chinense Var. Parviflorum. J Nat Prod.

[18] Zhang Y, Pizzute T, Pei M (2014). A review of crosstalk between MAPK and Wnt signals and its impact on cartilage regeneration. Cell Tissue Res.

[19] Sun HY, Hu KZ, Yin ZS (2017). Inhibition of the p38-MAPK signaling pathway suppresses the apoptosis and expression of proinflammatory cytokines in human osteoarthritis chondrocytes. Cytokine.

[20] Yoon YM, Kim SJ, Oh CD, Ju JW, Song WK, Yoo YJ, Huh TL, Chun JS (2002). Maintenance of differentiated phenotype of articular chondrocytes by protein kinase C and extracellular signal-regulated protein kinase. J Biol Chem.

[21] Linda T, Hideaki N (2012). Proteases involved in cartilage matrix degradation in osteoarthritis. Biochim Biophys Acta.

[22] Fawzy RM, Hashaadd NI, Namsorour AL (2017). Decrease of serum biomarker of type II collagen degradation (Coll2-1) by intra-articular injection of an autologous plasma-rich-platelet in patients with unilateral primary knee osteoarthritis. Eur J Rheumatol.

[23] Houard X, Goldring MB, Berenbaum F (2013). Homeostatic mechanisms in articular cartilage and role of inflammation in osteoarthritis. Curr Rheumatol Rep.

[24] Martin L, Richard FL (2012). Effects of aging on articular cartilage homeostasis. Bone.

[25] Mary BG (2012). Chondrogenesis, chondrocyte differentiation, and articular cartilage metabolism in health and osteoarthritis. Ther Adv Musculoskelet Dis..

[26] Dinesh B, Tatiana B, Mario N (2013). Current interventions in the management of knee osteoarthritis. J Pharm Bioallied Sci.

[27] Nahid A, Tariq MH (2012). Current nutraceuticals in the management of osteoarthritis: a review. Ther Adv Musculoskelet Dis.

[28] Zuscik MJ, Hilton MJ, Zhang X, Chen D, O’Keefe RJ (2008). Regulation of chondrogenesis and chondrocyte differentiation by stress. J Clin Invest.

[29] Mobasheri A, Bay-Jensen AC, van Spil WE, Larkin J, Levesque MC (2017). Osteoarthritis year in review 2016: biomarkers (biochemical markers). Osteoarthr Cartil.

[30] Eo SH, Choi SY, Kim SJ (2016). PEP-1-SIRT2-induced matrix metalloproteinase-1 and -13 modulates type II collagen expression via ERK signaling in rabbit articular chondrocytes. Exp Cell Res.

[31] Van Doren SR (2015). Matrix metalloproteinase interactions with collagen and elastin. Matrix Biol.

[32] Liu-Bryan R, Terkeltaub R (2015). Emerging regulators of the inflammatory process in osteoarthritis. Nat Rev Rheumatol.

[33] Emanuela R, Garret AF (2011). Prostaglandins and inflammation. Arterioscler Thromb Vasc Biol.

[34] Lucarini R, Bernardes WA, Ferreira DS, Tozatti MG, Furtado R, Bastos JK, Pauletti PM, Januário AH, Silva ML, Cunha WR (2013). In vivo analgesic and anti-inflammatory activities of Rosmarinus Officinalis aqueous extracts, rosmarinic acid and its acetyl ester derivative. Pharm Biol.

[35] Khojasteh A, Mirjalili MH, Hidalgo D, Corchete P, Palazon J (2014). New trends in biotechnological production of rosmarinic acid. Biotechnol Lett.

[36] Hur YG, Suh CH, Kim S, Won J (2007). Rosmarinic acid induces apoptosis of activated T cells from rheumatoid arthritis patients via mitochondrial pathway. J Clin Immunol.

[37] Han S, Yang S, Cai Z, Pan D, Li Z, Huang Z (2015). Anti-Warburg effect of rosmarinic acid via miR-155 in gastric cancer cells. Drug Des Devel Ther.

[38] Moon DO, Kim MO, Lee JD, Choi YH, Kim GY (2010). Rosmarinic acid sensitizes cell death through suppression of TNF-alpha-induced NF-kappaB activation and ROS generation in human leukemia U937 cells. Cancer Lett.

[39] Scheckel KA, Degner SC, Romagnolo DF (2008). Rosmarinic acid antagonizes activator protein-1-dependent activation of cyclooxygenase-2 expression in human cancer and nonmalignant cell lines. J Nutr.

[40] Youn J, Lee KH, Won J, Huh SJ, Yun HS, Cho WG, Paik DJ (2003). Beneficial effects of rosmarinic acid on suppression of collagen induced arthritis. J Rheumatol.

[41] Darling NJ, Cook SJ (2014). The role of MAPK signalling pathways in the response to endoplasmic reticulum stress. Biochim Biophys Acta.

[42] Shi J, Zhang C, Yi Z, Lan C (2016). Explore the variation of MMP3, JNK, p38 MAPKs, and autophagy at the early stage of osteoarthritis. IUBMB Life.

